# Association of Plasma Levels of Nitric Oxide Oxidative Metabolites with Acute Stroke in Patients Presenting to the Emergency Department of a Low-Middle Income Country

**DOI:** 10.1155/2019/9206948

**Published:** 2019-06-02

**Authors:** Shahan Waheed, Ayaz Ghouse Kalsekar, Ayeesha Kamran Kamal, Nathan S. Bryan, Asad I. Mian

**Affiliations:** ^1^Emergency Medicine, Aga Khan University Hospital, Stadium Road, Karachi 74800, Pakistan; ^2^Department of Pathology and Genomic Medicine, Houston Methodist Hospital, Houston, TX, USA; ^3^Section of Neurology, Department of Medicine, Aga Khan University Hospital, Karachi 74800, Pakistan; ^4^Department of Molecular & Human Genetics, Baylor College of Medicine, Houston, TX, USA

## Abstract

**Introduction:**

Acute stroke incites an inflammatory reaction in the brain's microvasculature, activating formation of nitric oxide oxidative metabolites, nitrate and nitrite (NOx, collectively), measurable in plasma. Our objectives were to investigate plasma NOx in patients with acute stroke presenting to the Emergency Department (ED) and to determine if it could (i) differentiate between ischemic and hemorrhagic stroke; (ii) predict clinical outcomes.

**Methods:**

A cross-sectional study was conducted in the ED of Aga Khan University Hospital, from January 1 to December 31, 2016. Participants were enrolled if they had clinical acute stroke with confirmatory brain imaging to differentiate between ischemia and hemorrhage. Clinical demographic information, ancillary blood, and diagnostic specimens were collected as per standard of care since the center follows stroke algorithmic guidelines. Plasma NOx analysis was performed using high performance liquid chromatography. Clinical outcomes were assessed using Barthel Index and Modified Rankin Score. Data was analyzed using SPSS 19 and expressed in medians with interquartile ranges. Nonparametric tests were applied for comparing among groups. Pearson's correlation was used to determine associations with aforementioned stroke severity and disability scales.

**Results:**

Seventy-five patients were enrolled, with median age of 57 years (IQR 47-66 years), 53 (71%) were males, and 46 (61%) had ischemic stroke. Overall, median NOx was 20.8 *μ*M (IQR 13.4-35.3); there was no statistically significant difference between NOx in ischemic versus hemorrhagic stroke (21.2 *μ*M vs. 17.9 *μ*M; p=0.2). However, there was a significant positive correlation between NOx levels and aforementioned acute stroke scales [r(73)=0.417, p=0.0001], for both.

**Conclusion:**

Although plasma NOx could not differentiate between ischemia and hemorrhage, higher levels of the biomarker did show associations with poststroke disability scales. Further study with more patients in a multicenter trial is warranted to establish the real biomarker potential of plasma NOx in acute stroke.

## 1. Background

Stroke as a major cause of morbidity and mortality accounts for 5.5 million deaths annually and 44 million disability adjusted life years [1, 2]. Limited numbers of tertiary care hospitals and weak healthcare infrastructure in low-middle income countries (LMICs) place patients with acute stroke in jeopardy if they are not managed in a timely manner [3, 4]. The utilization of cost-effective biomarker based point of care tests for acute stroke will provide timely care to patients presenting with acute stroke in the ED [5].

Nitric oxide (NO), a gaseous signaling molecule, has been implicated in pathophysiological pathways that accompany acute cerebrovascular events [6, 7]. Acute stroke incites an intense inflammatory reaction that results in activation of vascular processes such as inflammation, production of NO, reactive oxygen species, and reactive nitrogen species, generated as downstream metabolites of NO. All of these can cause ischemic brain injury by reacting with macromolecules [6–9]. Previous studies have emphasized the potential of NO-based oxidative metabolites nitrate and nitrite (collectively referred to as NOx) as biomarkers of ischemic stroke [10]. A study done in Hyderabad, India, revealed that, in acute stroke, plasma levels of NOx were significantly higher in patients versus controls (without stroke) [11]. Furthermore, there were correlations between serum NOx and diseased atherosclerotic arteries as a predictor of first ever or recurrent stroke [12–14]. However, association of plasma NOx levels with stroke subtypes (ischemic versus hemorrhagic) has not been studied.

In this study we wished to test the hypothesis that plasma NOx levels would help differentiate between type and severity of stroke. Our primary objective was to determine if plasma NOx could differentiate ischemic from hemorrhagic stroke in patients presenting to the ED. Our secondary objective was to determine if plasma NOx levels were predictive of clinical outcomes in patients with acute stroke.

## 2. Materials and Methods

### 2.1. Study Design and Setting

This was a cross-sectional study conducted in the ED of Aga Khan University Hospital (AKUH), Karachi, Pakistan, from January 01 to December 31, 2016.

### 2.2. Study Procedure

**Figure 1:**
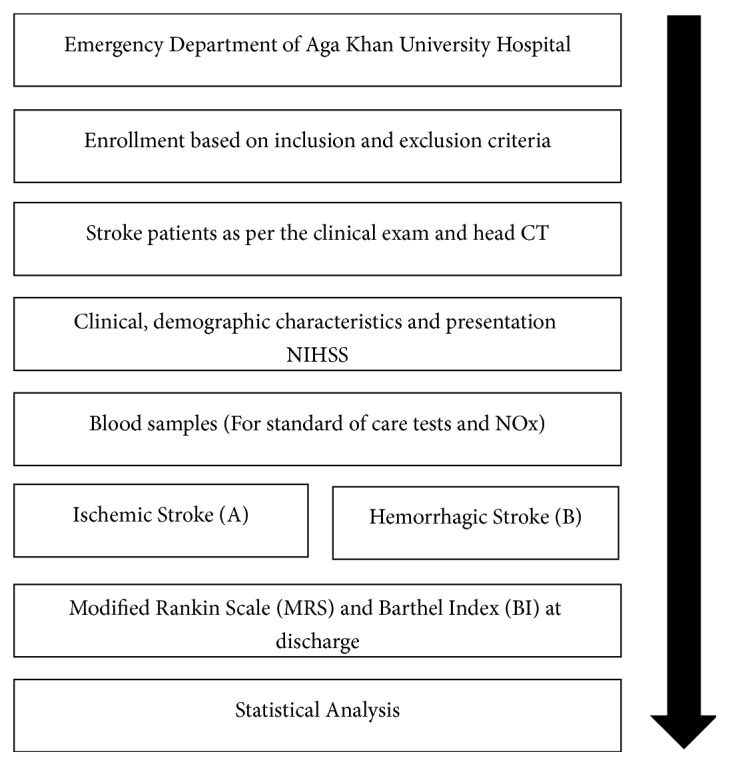
Study procedures.

The ED physician evaluated new onset stroke patients. The neurologic assessment included Glasgow Coma Scale (GCS) and National Institute of Health Stoke Scale (NIHSS). Ischemic and hemorrhagic stroke were differentiated on the basis of CT scan of the brain. The written consent was taken either from the patient or from the first-degree relative during blood sample collection for NOx assessment. The specimens to be assayed for NOx were frozen at -80°C and shipped to Baylor College of Medicine in Houston, Texas, for analysis by high performance liquid chromatography. The patients were followed on the neurology floor once admitted and at the time of discharge they were reevaluated on Modified Rankin Scale (MRS) and Barthel Index (BI) for functional disability that they might have sustained after the stroke event. The ethical review committee of the hospital approved the study (approval # 3045-EM-ERC-14). The study procedure is shown in Figure 1.

### 2.3. Sample Size Calculation

Based on the study by Rajeshwar* et al.* [11] and using WHO software for sample size calculation a difference in mean levels of p[NOx] with acute stroke subtypes of 3*μ*M, power of 80%, and type I error of 0.05, we calculated a sample size of at least 38 per group, respectively.

### 2.4. Patient Inclusion and Exclusion Criteria

Patients presenting to the ED were eligible for inclusion in the study if they fulfilled the following requirements:

(a) Either gender with age equal to or greater than 18 years

(b) Sudden onset of neurological deficit consistent with the World Health Organization criteria for stroke [15]

(c) First cerebrovascular accident only

(d) Evidence of stroke on MRI or noncontrast enhanced CT scan

(e) MRS less than 2 prior to the presenting stroke

Patients were excluded if they had transient cerebral ischemia or stroke events in case of blood disease (thrombocytopenia or coagulopathy) or brain tumors. Patients with any malignancy, presently suffering from pneumonia, alcohol or drug dependence, major cardiac, renal, hepatic, endocrinological disorders, skeletal disorders, and recent infections, were also excluded as those states have all been implicated, directly or indirectly, in modulating NO oxidative metabolite levels [11].

### 2.5. Plasma NOx Determination

Detailed analytical procedures for NOx analysis using high performance liquid chromatography (HPLC) have been previously described by Bryan and Grisham [16]. Briefly, prior to HPLC, in preparation for NOx analysis, methanol (1:1 v/v) was added to each plasma sample, vortexed and then centrifuged for 10 min to precipitate proteins. Each patient specimen was run in triplicate and the average was determined for further analysis. A dedicated ENO-20 HPLC System (EiCom Corporation, USA) was employed. This system was sensitive and selective for the measurement of nitrate and nitrite in all biological matrices with high throughput capacity. The ENO-20′s high sensitivity was attained by the combination of a diazo coupling technique with the extract to be measured and separation of nitrite and then nitrate using a reverse-phase column. To separate nitrite and nitrate, the nitrate was first reduced to nitrite through a reaction with cadmium and reduced copper inside a reduction column. The two resolved peaks were then mixed with Griess reagent (dinitrogen trioxide, N_2_O_3_, generated from acidified nitrite that reacts with sulfanilamide) in-line to form the classical diazo compound detected spectrophotometrically. This system allowed for easy sample preparation, little if any cross-reactivity and high throughput when coupled with an autosampler. The system was adaptable for a wide range of nitrite and nitrate concentrations regardless of their respective ratios and operated at a sensitivity level of 1nM × 100-*μ*L injections for each anion with no interference from protein or other colored species.

### 2.6. Statistical Analysis

SPSS v. 20 was used for statistical analysis. Patient demographic characteristics were described using frequencies for categorical variables and measures of central tendency and dispersion for continuous variables. Distributions were explored for plasma NOx concentrations and since they were found to be non-Gaussian in distribution, the nonparametric Mann-Whitney U test was used to make statistical comparisons across groups. Comparisons were made across group ischemia and hemorrhage stroke as well as among groups in nitrite, nitrate and NOx values. Pearson's correlation and regression analysis were performed between plasma NOx and GCS, NIHSS, MRS, and BI. P-value less than 0.05 was considered statistically significant.

## 3. Results

A total of 75 patients were enrolled in our study with a median age of 57 years (IQR 47-66 years). Forty-six (61.3%) had ischemic stroke and 29 (38.7%) hemorrhagic. There were 53 (70.7%) males and majority of the patients (n=38; 50.7%) presented in the morning. Among males, 32 of 46 (69.6%) had ischemic while rest (72.4%) had hemorrhagic stroke. Among females, 14 of 46 (30.4%) had ischemic stroke, while the rest (72.4%) had hemorrhagic stroke.  Table 1 demonstrates the demographic and clinical characteristics of patients on presentation to ED and on discharge.

### 3.1. Type of Stroke and Its Association with Stroke Outcome Measures

The evaluation of the type of stroke (ischemia versus hemorrhage) with clinical outcomes (GCS, NIHSS, MRS, and BI) was done to analyze the severity of stroke at presentation and discharge. In ischemia, 16 (34.8%) of stroke patients had a moderate GCS [9–12] at presentation whereas, in hemorrhage, it was 14 (48.3%); p-value 0.001. Presentation NIHSS in ischemia was 5-15 in 20 (43.5%) compared to 10 (34.5%) in hemorrhage; p-value 0.002. On discharge two scales were measured, namely, MRS and BI. As shown in Table 1, the MRS at discharge showed more disability in ischemic versus hemorrhagic stroke patients (14 (30.4%) ischemic vs. 11 (37.9%) hemorrhagic; p-value 0.005).

### 3.2. Plasma NOx and Its Association with Type of Stroke

**Figure 2:**
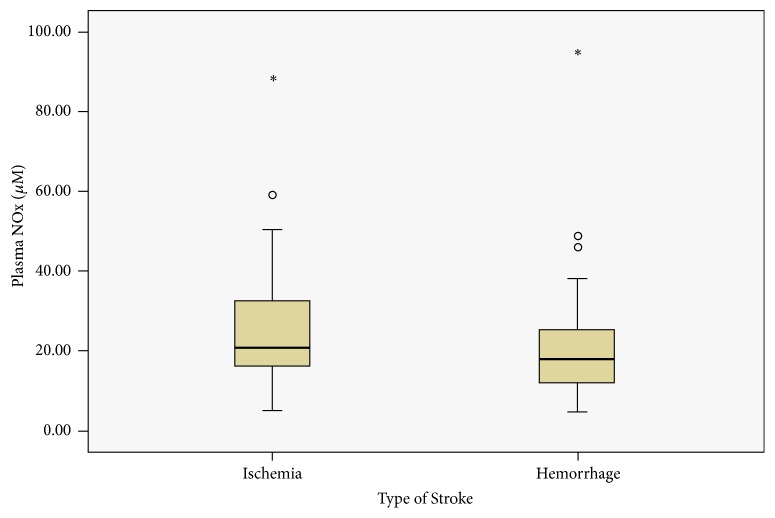
Absolute median NOx concentrations in patients presenting to the ED of Aga Khan University Hospital with ischemia or hemorrhage. Data are shown as box plots with the heavy line in the box representing the median (50^th^ percentile) and the ends of the box representing 25^th^ and 75^th^ percentiles, respectively. Groups were compared using the nonparametric Mann-Whitney U test (p=0.21).

In all stroke patients taken together, the median plasma nitrite concentration was 0.38 *μ*M (IQR 0.21, 0.71), nitrate 19.63 *μ*M (IQR 12.93,33.38), and NOx 20.8 *μ*M (IQR 13.4-35.3). Plasma NOx in ischemic versus hemorrhagic stroke is represented graphically in Figure 2. Although median plasma NOx was higher in ischemic stroke compared to hemorrhagic, the difference was not statistically significant (21.2 *μ*M vs. 17.9 *μ*M; p=0.2).

### 3.3. Plasma NOx and Its Association with Stroke Outcome Measures

There was no significant association between plasma NOx levels and GCS at presentation to the ED (r(73)=0.1; p=0.39). Nor was there any significant correlation between plasma NOx and NIHSS at presentation (r(73)=-0.042; p=0.72). However, there was a significant positive correlation between NOx levels and the two acute stroke disability scales measured at discharge from the hospital, namely, BI and MRS (r(73)=0.417; p=0.0001, for both).

## 4. Discussion

In this study we showed that in acute stroke patients presenting to the ED of an LMIC, plasma NOx levels showed a positive association with poststroke disability scales at discharge, namely, MRS and BI. Our study is unique in reporting this that has not been reported in previous studies [14–17]. This association may be explained by the disability that potentially became more pronounced with increasing inflammation as time passed.

NOx has shown an important role in homeostatic vasodilation and the regulation of blood flow and its level has been reported to be higher in the cerebrospinal fluid with early neurological deterioration, as may happen in stroke. The NOx metabolites are markers of ischemia and inflammation, and as such they may be useful in quick assessment and prognosis in stroke, as shown by Rajeshwar* et al.* [11], but their major limitation is specificity. The latter fact may be the reason why plasma NOx could not differentiate between ischemia and hemorrhage, as the etiologies of the stroke, in our study.

The plasma levels of the NOx subcomponents were higher in our study than published normal ranges in healthy adult subjects (1.84 *μ*M in our patient cohort versus 0.2-0.5 *μ*M) [17]. However, plasma nitrate concentration in our study subjects showed no difference as compared to published healthy adult range (24.7 *μ*M versus 20-40 *μ*M) [17, 18]. This is contrary to the results published by Serrano Ponz et al. that showed decrease concentrations in acute stroke patients [17]. Genetic makeup of population plays a role in plasma NOx production rate in the inflammatory pathway and accounts for different plasma NOx values among different population subsets [15]. Moreover, plasma NOx levels may be affected by age, gender, and dietary intake. A study done in Japan showed age-based variability in plasma NOx among healthy adults with increased NOx levels as the patient age from 20-60 years [15]. The values also showed variability with diet, as mentioned above, with a range of (43 versus 92 *μ*M) measured at fasting and after 150 grams of celery intake, respectively, reported in the same study [18].

The duration between onset of cerebrovascular insult and presentation to the ED might also influence NOx plasma concentrations. Since the plasma NOx levels rise as time passes and the duration from the onset of symptoms to the presentation to the ED as there are few stroke centers in the city, patients may present within different time scales that might result in increased NOx levels. This is contrary to the results published by Rajeshwar* et al.* that showed no association between NOx and type of stroke or outcome [11]. Our study results did show raised levels in acute stroke which are in accordance with the data published in previous studies. The regular measurement of NOx levels if showing steep rise might further pose an argument for its association with increased severity that is shown in previous studies. A major strength of our study is its prospective nature (with follow up). Second, the data set was evaluated for the first time in terms of NOx levels with respect to acute stroke in ED. Finally, there are no previous studies from Pakistan evaluating the association of NOx with poststroke disability scales.

Our study had several limitations. Dietary intake, especially if rich in NOx (as is the case in Mediterranean food), by impacting plasma NOx, could have potentially confounded our results. Although we were cognizant of this fact, in the acute care setting of the ED, it was not feasible for us to control for patients' dietary intake. The time of stroke onset and presentation to ED may explain the variability of plasma NOx levels as seen in our study. We were also limited by the small sample size of 75 patients. Furthermore, stroke subtypes were not evenly distributed, with only 29 of 75 patients (40%) presenting with hemorrhagic stroke in the study period of 12 months. The higher prevalence of ischemic stroke in our population subset may explain this discordance [2]. Khealani* et al.* report ischemic stroke as more common than hemorrhagic stroke due to increased risk contributed by high prevalence of hypertension, diabetes, and heart disease in our population [15].

## 5. Conclusion

Plasma NOx could not differentiate ischemic from hemorrhagic stroke patients presenting to the ED of an LMIC. Significant association was observed with higher levels of the biomarker and poststroke disability scales.

## Figures and Tables

**Table 1:** Demographic and clinical characteristics of patients with acute stroke on presentation to Emergency Department and on discharge from the Aga Khan University Hospital, Karachi, Pakistan, January 01 to December 31, 2016.

Variable	Total N (%)	Ischemia N(%)	Hemorrhage N(%)
Age in years: Median (IQR)	57(47-66)	58(49-70)	54(45-61)

*Gender*			
Male	53(70.7)	32(60.3)	21(39.6)
Female	22(29.3)	14(63.6)	8(36.3)

Stroke	75(100)	46(61.3)	29(38.7)

*Time of onset*			
Morning	38(50.7)	26(56.5)	12(41.4)
Afternoon	13(17.3)	7(15.2)	6(20.7)
Evening	13(17.3)	6(13)	7(24.1)
Overnight	11(14.7)	7(15.2)	4(13.8)

*GCS at presentation∗*			
15	22(29.3)	17(37)	5(17.2)
13-14	12(16)	11(23.9)	1(3.4)
9-12 <9	30(40) 11(14.7)	16(34.8) 2(4.3)	14(48.3) 9(31)

*NIHSS at presentation*			
Minor (1-4)	9(12)	9(19.6)	0
Moderate (5-15)	29(38.7)	20(43.5)	9(31)
Moderate/Severe (16-20)	24(32)	14(30.4)	10(34.5)
Severe (21-42)	13(17.3)	3(6.5)	10(34.5)

*Modified Rankin Scale at discharge*			
No disability (0)	2(2.7)	0	2(6.9)
Able to carry out everyday activities (1)	12(16)	11(23.9)	1(3.4)
Slight disability (2)	13(17.3)	10(21.7)	3(10.3)
Moderate disability (3)	18(24)	11(23.9)	7(24.1)
Moderate severe disability (4)	25(33.3)	14(30.4)	11(37.9)
Severe disability (5)	2(2.7)	0	2(6.9)
Dead (6)	3(4)	0	3(10.3)

*Barthel Index at discharge*			
Slight disability (91-100)	1(1.3)	0	1(3.4)
Moderate dependency (61-90)	8(10.7)	7(15.2)	1(3.4)
Severe dependency (21-60)	48(64)	30(65.2)	18(62.1)
Total dependency (0-20)	18(24)	9(19.6)	9(31)

*∗*GCS: Glasgow Coma Scale; ^∧^NIHSS: National Institute of Health Stroke Scale.

## Data Availability

The data used to support the findings of this study are available from the corresponding author upon request.

## Abbreviations

BI: Barthel Index
ED: Emergency Department
GCS: Glasgow Coma Scale
IQR: Interquartile range
LMICs: Low-middle income countries
MRS: Modified Rankin Scale
NIHSS: National Institute of Health Stroke Scale
NO: Nitric oxide
NOx: Nitric oxide oxidative metabolites.
